# Impact of the Integrated Program of Transdiagnostic Treatment and Parent Education on the Social Anxiety of Female Students

**DOI:** 10.7759/cureus.55299

**Published:** 2024-02-29

**Authors:** Sedigheh Pishdar, Solaleh Kalantari, Sara Kalantari, Hamid Reza Sheikhi, Zeinab Kuchaki

**Affiliations:** 1 Clinical Psychology, Islamic Azad University of Bandar Abbas, Bandar Abbas, IRN; 2 Personality Psychology, Islamic Azad University Sari Branch Campus, Mazandaran, IRN; 3 Family Therapy, University of Science and Culture, Tehran, IRN; 4 Department of Nursing, Islamic Azad University, Qaenat Branch, Qaenat, IRN; 5 Department of Nursing, Ilam University of Medical Sciences, Ilam, IRN

**Keywords:** combined program of transdiagnostic treatment and parent education, social anxiety, depression, parent-child relationship, parent education, up therapy

## Abstract

Objective: The objective of this study was to assess the efficacy of the combined program of transdiagnostic treatment and parent education in reducing social anxiety among female students.

Methodology: This descriptive cross-sectional study was conducted among all female elementary school students in Bandar Abbas, Iran, during the academic year 2022-2023. The social phobia questionnaire was given to all female students in grades three to six to assess individuals in terms of the social anxiety disorder (SAD) variable. We used the social anxiety scale developed by Leibovitz as the questionnaire in this investigation. This self-assessment questionnaire was designed for individuals aged 18 and above. It consists of 24 statements, divided into two subscales: performance anxiety (13 statements) and social settings (11 statements). Each item is individually assessed for fear intensity on a scale of 0 to 3, ranging from no to extreme. Similarly, avoidance behavior is evaluated on a scale of 0 to 3, representing the frequency ranging from never to always.

Results: The mean general anxiety levels among both groups (students vs. parents) during the pre-test were similar (48.06 ± 4.39 vs. 48.06± 4.1). However, in the post-test, the mean of the experimental groups was lower than that of the pre-test (32.13 ± 3.77 vs. 47.2 ± 3.6). The normality assumption for the pre-test and post-test variables of generalized anxiety was verified with a significance level over 0.05 (p ≥ 0.05).

Conclusion: The findings demonstrated that the integrated meta-diagnostic treatment program for parents had a more pronounced effect on alleviating their social anxiety in comparison to students. These findings imply that if parents possess a comprehensive understanding of the factors contributing to their children's anxiety, it will significantly enhance their ability to mitigate their child's social anxiety.

## Introduction

Anxiety disorders are the most common form of emotional disturbance among children and adolescents, with a prevalence rate that varies from 5% to 10% [1,2]. The incidence of depressive disorders is slightly lower, ranging between 3% and 5% [1]. Comorbidity, which refers to the coexistence of many medical diseases in an individual, is frequently observed among teenagers, with a notably high prevalence of comorbidity between anxiety and depression. Young individuals with an anxiety or depressive disorder are roughly 30 times more likely to have the other disorder compared to their counterparts without anxiety or depression [3]. The simultaneous presence of multiple medical illnesses has profound implications on the intensity of symptoms and the general state of well-being [4]. The simultaneous presence of anxiety and depression in children and adolescents leads to heightened symptom severity, increased psychological discomfort, disruption in everyday functioning, and a poorer long-term prognosis, as compared to those who only have one of these conditions [4,5]. High levels of anxiety significantly raise the probability of experiencing depression during the early stages of life [6]. Research reported that co-occurrence of these two illnesses may indicate an increased genetic predisposition [7]. By contrast, specific studies have suggested that the presence of emotional disorders can impact an individual's response to treatment [8,9]. Although there is a lack of extensive research on the subject, various tests have demonstrated that adolescents who have major depression (i.e., when depression is the dominant disease) tend to have poorer treatment outcomes when they also have an additional anxiety disorder [10,11]. Only a small number of literary works emphasize the consistent evidence of a mutual connection between coexisting depression and treatment outcomes for underlying anxiety. Nevertheless, data indicate that children and adolescents who have both anxiety and depression are more likely to have significant challenges even after undergoing treatment [8,9]. 

There exist notable clinical and causal resemblances between anxiety and depression in young individuals, which can be primarily ascribed to shared underlying mechanisms [12,13]. These pathways may involve a mix of hereditary sensitivity and a common tendency toward increased emotional reactivity, more intense negative views about the world, and avoidance measures to reduce the activation of unpleasant emotions [14]. As a result of these standard features, the focus has turned to adopting transdiagnostic treatments, which mainly address symptoms or widespread processes across different diseases [12-13,15]. These courses aim to target underlying mechanisms that are common to both anxiety and depression, such as cognitive distortions, behavioral avoidance, deficient problem-solving, or insufficient coping skills. Thus far, there has been a relatively small amount of scientific evaluation carried out on these activities that focus on young people.

Some early studies recorded the successful handling of adolescents who had both anxiety and depression, portraying them as clinical case reports or limited uncontrolled assessments [16,17]. Recently, a small number of randomized clinical trials have emerged, assessing treatments that address both anxiety and depression in various diagnostic categories [15,18-19]. Furthermore, these trials have incorporated a minority proportion of people who had additional comorbidities. Overall, these trials have shown promising results, although there has been significant variation in the level of advancement [19-21]. Therefore, our study is the initial endeavor to autonomously assess the efficacy of a particular transdiagnostic treatment for anxiety disorders, emphasizing its strengths and weaknesses. Our objective is to gather empirical evidence that supports the existence of a hidden nuclear mechanism in the extradiagnostic treatment of bottom-up therapy (UP therapy). The notable disparities identified in the effect sizes of anxiety when compared, which are considered distinct outcomes, may suggest that there is no singular model of susceptibility based on a single factor.

## Materials and methods

This descriptive cross-sectional study evaluated the efficacy of the integrated program combining transdiagnostic treatment and parent education in reducing social anxiety among female students from 2022 to 2023 after obtaining ethical approval from Islamic Azad University (approval no. IAU/5723). In the current study, the effectiveness of the extradiagnostic treatment of UP was evaluated using a semi-experimental design, which included a pre-test and post-test with a control group. The target group for this study consisted of all female elementary school students in Bandar Abbas, Iran, during the academic year 2022-2023. A total of 30 female kids from the third to sixth grades of an elementary school in Bandar Abbas City were selected to achieve the research objective. These students achieved the highest scores in the initial administration of the social phobia questionnaire. The selection process involved employing the multi-stage cluster sampling and screening approach. We selected seven schools from 60 elementary schools for girls using a random sampling technique. From each of these schools, one class was randomly picked from the grades ranging from third to sixth. The social phobia questionnaire was given to all female students in grades three to six to assess individuals in terms of the social anxiety disorder (SAD) variable. From this initial sample, individuals who obtained the highest score on the social anxiety questionnaire achieved a score of 50, indicating a significant level of social anxiety. Following the clinical interview, 30 female students were randomly divided into experimental and control groups, comprising 15 individuals each. A total of 14 instructional sessions were conducted each week, with two 60-minute sessions running simultaneously for both children and parents.

The social anxiety scale developed by Leibovitz was employed as the questionnaire in this investigation [22]. It consists of 24 statements, divided into two subscales: performance anxiety (13 questions) and social settings (11 questions). Each item is individually assessed for fear intensity on a scale of 0 to 3, ranging from no to extreme. Similarly, avoidance behavior is evaluated on a scale of 0 to 3, representing the frequency ranging from never to always. Thus, this examination provides an overall assessment of social anxiety and individual scores for performance phobia, performance-avoidance, social phobia, and social avoidance. The entire execution of this questionnaire requires approximately 30 minutes. Table 1 represents the reliability of the questionnaire we used for our study. Then, relevant methods of descriptive statistics, such as frequency, percentage, central tendency indices, dispersion, and distribution, were employed to describe the data based on the variables analyzed. Furthermore, column charts were utilized to represent the data graphically. Lastly, the covariance analysis test was employed to address the study hypotheses, considering the nature of the data.

**Table 1:** Content of treatment in the experimental group.

Meetings	Content of children's educational sessions	Content of parent training sessions
First session	Getting to know each other, learning different parts of an emotion, doing useful and non-useful things when experiencing an emotion	Familiarity with the structure of this treatment and clue skills, education about the three-part model of emotion, alienation cycle, and emotional behaviors
Second session	Talk about experiencing different things, three parts of one thrill, rewards for new and brave behaviors	Emotion worksheet training, four common emotional educational behaviors, and opposite educational behaviors
Third session	Aversive behaviors, steps of a scientific experiment, performing activities as aversive behavior when experiencing emotion	Using scientific experiments to challenge behavior, reinforce positive behaviors, and try to adapt
Fourth Session	Body clues, body scanning skills	Learning how to experience emotions in children, body scanning, sensory exposure, empathizing with the child's efforts
Fifth session	Identifying stereotyped judgments, intellectual traps	Learning flexible thinking, mental traps, reinforcement and punishment, emotional educational behavior and conflicting educational behavior
Sixth session	Using detective thinking and problem-solving	The purpose of detective thinking, over-controlling and over-supporting behaviors, practice of giving independence to the child
Seventh session	Experiencing the thrills here and now	Problem-solving skills and detective thinking
Eighth session	Thriller detective skills review, science experiment game	The importance of experiencing excitement, practicing present moment awareness, non-judgmental awareness
Ninth session	A scientific experiment to deal with strong emotions, protective behaviors	Facing emotional situations, modeling healthy emotions, completing the child's emotional behavior form
Tenth session	Ladder of excitement, exposure	Supporting child exposure practice, use of emotion ladder, safety behaviors
11th to 14th sessions	Review skills in the Thrill Detectives program, practice with parents, celebrate becoming a Thrill Detective	Management of common challenges during exposure, follow-up form for exposure to situational emotions, planning for continued improvement, slippage vs. relapse.

Statistical analysis

For the statistical analysis, IBM SPSS Statistics for Windows, version 23 (released 2015; IBM Corp., Armonk, New York, United States) was used for testing the analysis. The statistical analysis was conducted by assessing the assumptions of covariance analysis, including the homogeneity of regression coefficients within groups, homogeneity of error variance within groups, linearity of the dependent variable and pre-test variable, normality of the dependent variable, and independence of the data points. 

This study aimed to examine the impact of the independent variable (an integrated program of a metadiagnostic treatment and parent-child education) on the dependent variable (generalized anxiety). A multivariate covariance analysis statistical test was employed to find the impact of independent and dependent variables. This test helped control for the variance caused by pre-tests, which measured the initial differences between the subjects in the two groups. A p-value less than 0.05 was considered for statistical significance.

## Results

Table 2 provides a concise overview of the descriptive statistics for the generalized anxiety variable in both the control and experimental groups. The mean levels of general anxiety among both groups during the pre-test were similar (48.06 ± 4.39 vs. 48.06± 4.1) However, in the post-test, the mean of the experimental groups was found to be lower than that of the pre-test (32.13 ± 3.77 vs. 47.2 ± 3.6). The various measures of central tendency and dispersion indicate that the distribution of the participants' scores in all variables and stages closely approximates a normal distribution. In order to assess normality, the data analysis part also included the implementation of the Kolmogorov-Smirnov test.

**Table 2:** Summary of the descriptive statistics of general anxiety variable in the pre-test and post-test of different groups

Level	Group	Total number	Mean ± SD	Elongation	Elongation error	Crookedness	Minimum	Maximum
Pre-test observations	Experiment	15	48.06 ± 4.39	-1.045	1.091	0.391	41	51
	Control	15	48.06± 4.1	-1.050	1.014	0.327	42	56
Post-test observations	Experiment	15	32.13 ± 3.77	-1.289	1.091	0.314	27	38
	Control	15	47.2 ± 3.6	-0.699	1.041	0.474	43	56

As shown in Table 3, the assumption of normality in the pre-test and post-test variables of generalized anxiety was confirmed with a significance level greater than 0.05 (p ≥ 0.05).

**Table 3:** Kalmgrove-Smirnoff test to check the normality of generalized anxiety variables in the control and experimental groups

Level	Group	Statistics	Degree of freedom	p-value
Pre-test observations	Experiment	0.173	12	0.212
	Control	0.184	12	0.169
Post-test observations	Experiment	0.181	12	0.196
	Control	0.129	12	0.210

Table 4 shows that Levin's F test was used to assess the equality of variances in two groups for the general anxiety variable related to parent and child impact. The test yielded F_1,28 = 0.384, indicating a lack of significant difference in variances between the groups. The significance level was found to be 0.012, which is lower than the threshold of 0.05, providing validation of the assumption of equal variances.

**Table 4:** Levin's F test to check the assumption of equality of variances in the control and experimental groups

Level	Degree of freedom 1	Degree of freedom 2	F	p-value
Generalized anxiety (child)	1	28	0.384	0.012
Generalized anxiety (parent)	1	28	1.168	0.010

The variance test (Pillai effect) was conducted to examine the slope of the regression in the parent and child efficacy widespread anxiety variable in two groups. The results, shown in Table 5, verified the test with F_((2,4)) = 1.80 and a significance level of 0.144 (p ≥ 0.05).

**Table 5:** Variance test to check the regression slope in the two groups

Level	Degree of freedom	Amount of	F	p-value
Generalized anxiety (child)	2	0.060	0.74	0.488
Generalized anxiety (parent)	2	0.150	2.04	0.154
Group	2	0.137	1.82	0.183
Group pre-test	2	0.26	1.80	0.144

The Mbox test was conducted to assess the equality of variance-covariance matrices in two groups, as indicated in Table 6. The test yielded a p-value of 0.082, which is greater than the significance level of 0.05, confirming the assumption of equality of variance-covariance matrices.

**Table 6:** Mbox test to check the assumption of equality of covariance matrices in two groups

Mbox test	F	Degree of freedom 1	Degree of freedom 2	p-value
7.255	2.231	3	14112000	0.082

The results in Table 7 indicate that a multivariate covariance analysis (Pillai effect test) was conducted to compare the adjusted scores of parent and child affective generalized anxiety between the two groups. The analysis revealed a significant difference between the two groups, with an F-value of 38.42 (2.25) and a significance level of 0.001. A statistically significant p-value of ≤0.01 was found when evaluating the impact of the combined linear effect of the generalized anxiety variable on the efficacy of parents and children in both the experimental and control groups.

**Table 7:** Results of the multivariate covariance analysis (Pillai effect test) between the two groups on the adjusted scores of generalized anxiety

Level	Degree of freedom	Amount	F	p-value	Effect size
Generalized anxiety (child)	2	0.507	12.74	0.001	0.507
Generalized anxiety (parent)	2	0.434	9.03	0.001	0.434
Group	2	0.137	38.82	0.001	0.755

## Discussion

The purpose of the current study was to examine the impact of a comprehensive program combining a metadiagnostic treatment and parent education on the anxiety levels of female students. Anxiety disorders are prevalent among children and are the most common mental diseases in this age group. Identifying these problems at an initial stage can help mitigate their long-term consequences [8]. In recent years, there has been a significant focus on integrating intervention approaches for the treatment of mental diseases. Their focus lies on standard transdiagnostic psychological processes, similar to a later paradigm for common mental diseases [23]. As stated by Ahlen et al. [24], psychological processes can be classified as "transdiagnostic" if they meet the following criteria: (1) they can be assessed in both nonclinical and clinical samples, (2) they present across different diagnostic categories, and (3) they are involved in the development and persistence of many types of diseases. Notify A substantial collection of protocols known as a metadiagnostic has been created to target these processes in therapies specifically [25]. Using standard protocols that focus on treating a specific condition can cause doctors to face problems related to time and expense [23]. Norton's narrative review [26] and recent meta-analytic studies [27,28] provide compelling evidence supporting the efficacy of transdiagnostic therapy in alleviating symptoms of anxiety and sadness. Transdiagnostic techniques have shown efficacy in reducing the severity of comorbid anxiety disorders and anxiety symptoms, surpassing control conditions in general.

Furthermore, transdiagnostic therapies strongly correlate with elevated client satisfaction, a favorable therapeutic connection, and optimistic therapeutic expectations [29]. Our results are also consistent with these findings. In the current study, findings indicated that the integrated metadiagnostic treatment program for parents had a more significant effect on reducing their social anxiety compared to students. Results suggest that when parents are well-informed about the factors contributing to their children's anxiety, it can significantly diminish their child's social anxiety. Nonetheless, the comprehensive intervention program for youngsters, which includes additional therapeutic measures, has proven to be efficacious in mitigating social anxiety. The test results have demonstrated a notable decrease in anxiety levels.

During the pre-test period, the degree of pervasive anxiety in both the experimental and control groups in our study was similar. However, the post-test results indicate a notable decrease in general anxiety levels within the experimental group as compared to the control group. Comparing our results with previous meta-analyses of eight trials, both published and unpublished, the authors discovered a substantial moderate effect size (d = 1.29) for uncontrolled pre-post therapy aimed at reducing anxiety. In addition, they observed a modest effect size favoring transdiagnostic interventions over waiting. 

Nevertheless, it is essential to exercise caution when interpreting these findings due to methodological constraints, such as limited test subject sample size and a lack of research involving comparison groups [30]. Concerning the alteration and enhancement of the anxiety scores of the students involved in this study, we observed that treatment sessions impart specific techniques or skills. During therapy sessions, these students endeavor to comprehend the patterns of emotional avoidance, familiarise themselves with its influence on their emotional experiences, grasp the conflicting consequences of this avoidance, and substitute incompatible ways with adaptive strategies for regulating their emotions. Nevertheless, the meta-analyses conducted by Norton and Phillip [26] and Reinholt and Krug [28] concentrated explicitly on self-report measures of anxiety and conventional cognitive-behavioral therapy (CBT) interventions administered in either group or individual settings.

In contrast to previous works, Newby et al. [27] expanded and revised the existing scientific literature on the efficacy of various transdiagnostic CBT interventions. They conducted a comprehensive analysis of over 10 metadiagnostic methodologies that were implemented in multiple formats, such as telephone-based and Internet/web-based. They observed a significant overall effect size in favor of transdiagnostic techniques for the treatment of anxiety (pre-post = 0.86; gRCT = 0.65) and depression (pre-post = 0.91; gRCT = 0.80) in both pretreatment data (uncontrolled effect size) and randomized controlled trials (RCTs). Furthermore, in their study, the rates of the metadiagnostic treatment remained high at three and nine months after the treatment (uncontrolled effect size, with an anxiety score of 0.87). Subsequent research has verified the significant impact of transdiagnostic techniques in addressing anxiety and depression in adults, with an effect size of approximately 1.00.

In addition, a modest effect size of 0.50 was observed in children and adolescents [29]. Our research also shows that encouraging children to face emotional situations instead of avoiding them, promoting independence, and maintaining high expectations all contribute to positive parenting behaviors that effectively improve parents' ability to raise their children and their level of involvement with them. The customized parental training program provides instruction on recognizing and managing emotions in different situations, especially when dealing with children and teaching them how to be excellent parents; it improves mothers' parenting skills.

Some limitations of this research include the narrow selection of participants from a single institution for girls, the restriction to a specific academic year, and the small geographical region of primary and Bandar Abbas City. It is advisable to carry out comparable studies in several areas of the country, with boys from diverse age categories, to enhance the reliability of the findings when making generalizations. This program is capable of simultaneously utilizing the presence of parents. Future studies should explore the functional differences between fathers and mothers. Furthermore, child specialists can use these interventions to enhance children's ability to regulate their emotions and improve parents' parenting methods, considering the treatment's efficacy.

## Conclusions

The findings demonstrated that the integrated metadiagnostic treatment program for parents had a more pronounced effect on alleviating their social anxiety in comparison to female students. These results imply that if parents possess a comprehensive understanding of the factors contributing to their children's anxiety, it will significantly enhance their ability to mitigate their children's social anxiety. Nonetheless, the total intervention program for children, which includes additional therapeutic measures, has proven to be efficacious in diminishing social anxiety. The test outcomes have demonstrated a noticeable decrease in anxiety levels.
